# ACP-GBDT: An improved anticancer peptide identification method with gradient boosting decision tree

**DOI:** 10.3389/fgene.2023.1165765

**Published:** 2023-03-29

**Authors:** Yanjuan Li, Di Ma, Dong Chen, Yu Chen

**Affiliations:** ^1^ College of Electrical and Information Engineering, Quzhou University, Quzhou, China; ^2^ College of Computer, Hangzhou Dianzi University, Hangzhou, China; ^3^ College of Information and Computer Engineering, Northeast Forestry University, Harbin, China

**Keywords:** anticancer peptides, protein identification, biological sequence analysis, machine learning, artificial intelligence

## Abstract

Cancer is one of the most dangerous diseases in the world, killing millions of people every year. Drugs composed of anticancer peptides have been used to treat cancer with low side effects in recent years. Therefore, identifying anticancer peptides has become a focus of research. In this study, an improved anticancer peptide predictor named ACP-GBDT, based on gradient boosting decision tree (GBDT) and sequence information, is proposed. To encode the peptide sequences included in the anticancer peptide dataset, ACP-GBDT uses a merged-feature composed of AAIndex and SVMProt-188D. A GBDT is adopted to train the prediction model in ACP-GBDT. Independent testing and ten-fold cross-validation show that ACP-GBDT can effectively distinguish anticancer peptides from non-anticancer ones. The comparison results of the benchmark dataset show that ACP-GBDT is simpler and more effective than other existing anticancer peptide prediction methods.

## 1 Introduction

Cancer, one of the deadliest diseases in the world, kills millions of people every year (Hani et al., 2021). The development of anticancer drugs with high effectiveness and low side effects is gaining more attention. The traditional cancer treatment strategy is chemotherapy, but the shortcomings of chemotherapy drugs are high costs and side effects (Sun et al., 2022). Compared with traditional chemotherapy, anticancer drugs can effectively kill cancer cells and have low side effects. Therefore, researchers have put more effort into identifying and designing new anticancer drugs (Yang et al., 2023).

In recent decades, peptides with anticancer activity have become a potential alternative for cancer treatments. Compared with traditional chemotherapy, peptide-based therapy has many advantages, such as high tumor penetration, high specificity, low production cost, and ease of synthesis and modification. Anticancer peptides (ACPs) are short peptides with anticancer properties, consisting of 10–50 amino acids. They are molecular polymers between proteins and amino acids, consisting of dozens of amino acids connected by peptide bonds (Dong et al., 2021; Huo et al., 2022). ACPs have been widely studied as one of the most reliable anticancer drugs. They can kill cancer cells without destroying normal human cells. Based on these benefits, ACPs are increasingly used in clinical trials. Therefore, their accurate identification is important for exploring their action mechanism and developing therapeutic varieties.

Experimental methods for identifying ACPs are usually time-consuming and prohibitively expensive. Machine learning methods are completely based on sequence information and zero-cost. In recent years, many anticancer peptide recognition methods based on machine learning have been developed, including AntiCP, Hajisharifi’s model, iACP, ACPred-FL, PEPred-Suite, ACPred-Fuse, DeepACP, and ACP-MCAM. AntiCP (Tyagi et al., 2013) consists of three models. The first is AntiCP-ACC, which encodes amino acid sequences with amino acid composition and trains by using a support vector machine. The second model is AntiCP-DC, which uses dipeptide composition as a characteristic of peptide sequences and uses a support vector machine to train. The third model is AntiCP-BP, which is a binary profile-based support vector machine model. Hajisharifi et al. (2014) constructed an ACP classification model using PseACC as a feature representation method and SVM with a local alignment kernel. This method claims 83.82% accuracy on the built-in dataset. Wei Chen et al. (2016) proposed the ACP predictor called iACP, which represents peptide sequences with pseudo-amino acid composition with a G-gap dipeptide mode and uses SVM as the classifier. Wei et al. (2018) developed the ACP predictor ACPred-FL. This developed an effective feature-representation learning model, which can automatically extract the features from a pool of models, which is constructed on peptide sequence-based feature descriptors using an SVM algorithm. They subsequently proposed an improved predictor named “PEPred-Suite,” which can predict eight therapeutic peptides with different functions, including ACPs (Wei et al., 2019). Rao et al. (2020) developed a predictor called “ACPred-Fuse,” which can automatically identify whether a peptide sequence has anticancer efficacy. The feature representation learning model, which can use a variety of multi-view information, is first established. The random forest is then trained on these multi-view features. Yu et al. (2020) proposed a computational approach to identifying ACPs named DeepACP based on deep learning. They systematically compared the performance of three main deep learning architectures—recurrent, convolutional, and convolutional–recurrent neural networks—to distinguish ACPs and non-ACPs. They concluded that a recurrent neural network with bidirectional long short-term memory cells performs best. Wu et al. (2022) introduced a tool for predicting ACPs based on a multi-kernel CNN and attention model—ACP-MCAM. It can automatically extract effective information from the amino acid sequence and obtain feature encoding on node features and sequence features. Experimental results show that the model performs better than the other models mentioned previously. Although the existing predictors have made a lot of progress, their overall prediction accuracy is still not adequate for actual treatment application. In this study, the novel predictor ACP-GBDT is proposed, which encodes the peptide sequence with the merged-features consisting of SVMProt-188D and AAIndex descriptors. The merged-features show strong specificity and can distinguish anticancer from non-anticancer peptides. In our method, a gradient boosting decision tree (GBDT) was used to establish the identification model. Experimental results show that our method is more concise, efficient, and achieves better performance than the previously published methods.

## 2 Materials and methods

### 2.1 Framework of ACP-GBDT

**FIGURE 1:**
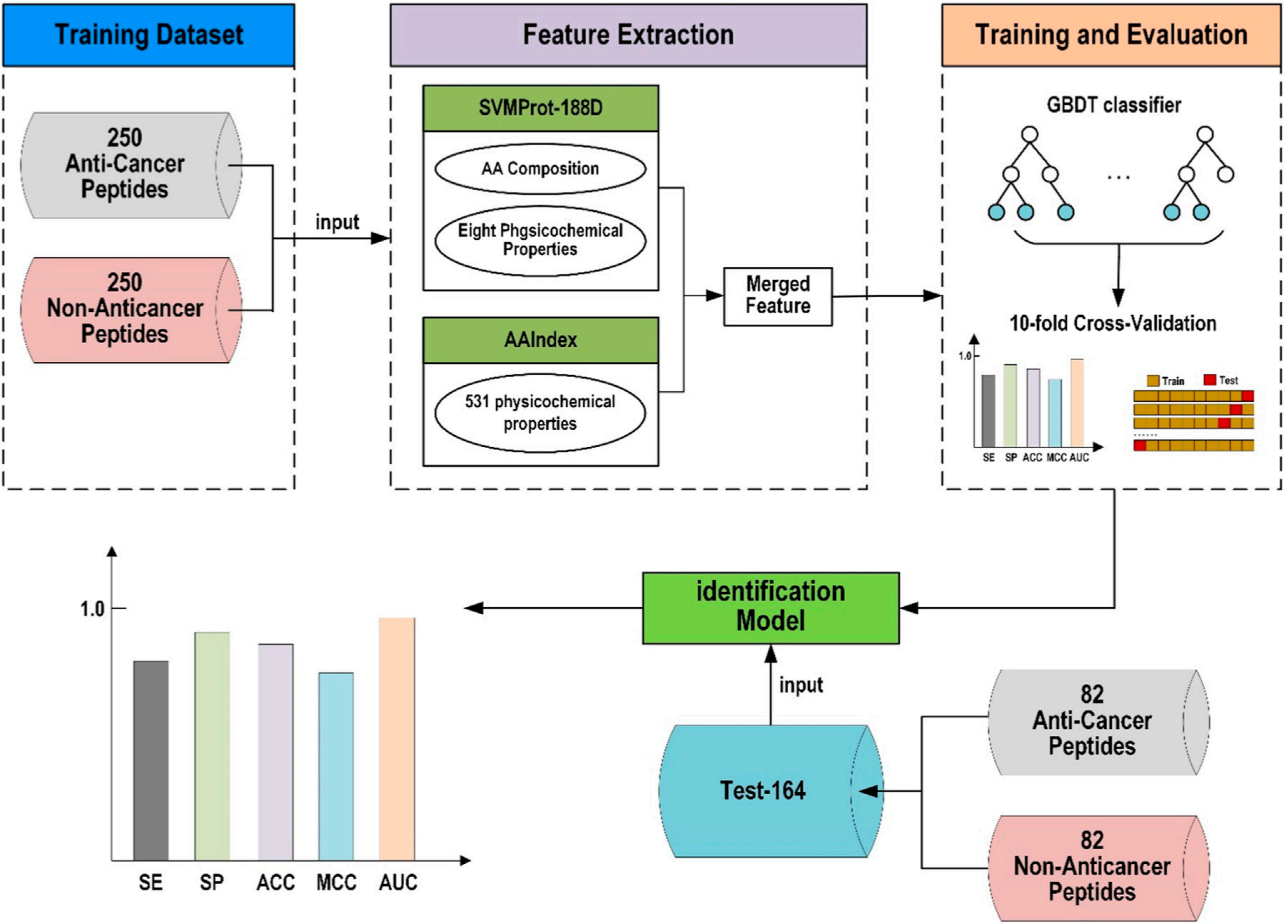
Framework of ACP-GBDT.

The framework of this study’s proposed anticancer peptide predictor based on gradient boosting decision tree (ACP-GBDT) is shown in Figure 1. First, the dataset used in this paper is the same as the ACPred-FL method. Second, the SVMProt-188D and AAIndex descriptors are extracted from the peptide sequences and merged to be used as the input of the classifier. Finally, a GBDT is used as the classifier to identify anticancer and non-anticancer peptides.

### 2.2 Dataset

This study directly used the datasets proposed in ACPred-FL. These were collected and collated by Leyi Wei et al. from three main resources: Tyagi et al., Chen et al., and a public ACP database, CancerPPD (Tyagi et al., 2015). The initial extracted positive samples include 3,212 ACPs. The negative samples include 2,250 non-ACPs extracted from CAMP, APD, and DADP. The homological bias of sequences can influence performance, so all samples included positive and negative examples filtered by CD-HIT (Li and Godzik, 2006; Wei et al., 2022) with the threshold 0.9. Samples with a similarity of more than 90% are thus removed. The final samples include 332 positive and 1,032 negative examples. To ensure the balance of datasets, 332 non-anticancer peptides were randomly selected from negative datasets. The selected samples were divided into a training set and a test set. The training set (Train-500) consisted of 250 anticancer and 250 non-anticancer peptides; it was used to train the model evaluating by ten-fold cross-validation. The test set (Test-164) consisted of 82 anticancer and 82 non-anticancer peptides and was employed to measure the generalization ability of the model’s evaluation by independent validation.

### 2.3 Feature extraction

In building a peptide predictor, feature extraction is the first and most significant step (Yones et al., 2021; Ao et al., 2022a; Yan et al., 2022). A feature with strong identification and high specificity can effectively differentiate positive from negative samples, thus greatly improving the performance of the predictor. We represented the peptide sequence with a merged-feature model, composed of the AAIndex descriptor (Peng and Zhu, 2022) and SVMProt-188D (Jiao et al., 2021). The merged-feature can encode peptide sequences from a different perspective and has some achievement in bioinformatics. SVMProt-188D encodes peptide sequences based on the frequency of 20 different amino acid compositions of the peptide sequence and eight physical and chemical characteristics. SVMProt-188D has effectively predicted diabetic protein markers (Qu et al., 2021) and antioxidant proteins (Caa et al., 2020). AAIndex encodes peptide sequences based on the AAIndex database, which consists of many physical and chemical properties of amino acids. Each amino acid is encoded into the numerical values according to its physicochemical properties. AAIndex has had great success in some bioinformatics problems such as predicting tumor T-cell antigens (Herrera-Bravo et al., 2021) and FAD binding sites (Ho et al., 2021). In this study, SVMProt-188D and AAIndex are first extracted from peptide sequences. Then, SVMProt-188D and AAIndex are merged. Finally, this merged-feature model is input into the classifier to predict whether a peptide is anticancer or non-anticancer. The experimental results are shown in Section 3.1. A detailed description of SVMProt-188D and AAIndex is given in the following sections.

#### 2.3.1 SVMProt-188D

According to the amino acid composition and the physical and chemical properties of peptide sequences, SVMProt-188D encodes peptides as a 188-dimensional vector. SVMProt-188D thus consists of two parts. One describes the frequency of 20 different amino acids in the peptide sequence, obtaining a 20-dimensional feature vector. The other part describes the eight physicochemical properties of amino acids, each property corresponding to 21 characteristic values. A 168-dimensional feature vector is thus obtained.

The first 20-dimensional feature vector corresponding to the composition information of amino acids is defined as

$$\left(v_{1},v_{2},...,v_{20}\right)=\frac{N_{i}}{L},$$ (1)

where *N*_*i*_ denotes the number of the *i*th amino acid in the peptide sequence and *L* denotes the total number of amino acids in the peptide sequence. Thus, $\sum v_{i}=1$ .

The latter 168-dimensional feature vector is related to eight physicochemical properties: secondary structure, hydrophobicity, charge, polarizability, normalized van der Waals volume, solvent accessibility, polarity, and surface tension. Each physicochemical property is classified into three groups, and the 21 characteristic values will be calculated according to these three groups for each physicochemical property.

A 21-dimensional feature vector is obtained for each physicochemical property. In detail, using hydrophobicity as an example, Group-1 is named “hydrophilicity,” Group-2 is “hydrophobicity,” and Group-3 is named “neutrality”. The frequency of amino acids belonging to each group is computed, obtaining the three-dimensional feature vector. Considering the situation of one amino acid in a group followed by another amino acid in another group—such as the transition from hydrophilicity to hydrophobicity, from hydrophilicity to neutrality, or from hydrophobicity to neutrality—the frequency is computed, and another three-dimensional feature vector is obtained. By calculating the proportions of the first, n*25%, n*50%, n*75%, and the last amino acids in the peptide sequence, a 15-dimensional feature vector is obtained. Therefore, a 21-dimensional feature vector is obtained for each physicochemical property. A 168-dimensional feature vector can be constructed by considering the eight physicochemical properties. Therefore, the 188-dimensional feature vector is obtained.

#### 2.3.2 AAIndex descriptor

Each amino acid has its own various properties. The combination of properties is specified by the genetic code, which is responsible for the diversity and specificity of biological functions and protein structure. Amino acids are the components of protein and have different characteristics in volume, shape, and chemical reactivity. Much theoretical and experimental research has been conducted to describe the biochemical and physicochemical properties of a single amino acid. The derived property is usually termed “amino acid indices,” which is a vector consisting of 20 numerical values. A total of 222 amino acid indices have been collected from the literature by Nakai et al. (1988), and their interrelationships have also been investigated using hierarchical cluster analysis. Tomii and Kanehisa (1996) not only extended the size of the collection to 402 amino acid indices but also collected 42 amino acid mutation matrices from research papers. Based on this, the AAIndex database was established by Nakai et al. and then developed by Tomii and Kanehisa.

A numerical value is given for each amino acid according to each physicochemical property. This means that there is a one-to-one correspondence between the 20 amino acids and numerical values. The current AAIndex database includes 544 physicochemical properties (Kawashima and Kanehisa, 2000). After removing the physicochemical properties with value “NA,” 531 physicochemical properties remain. The AAIndex is usually used to represent peptides of equal length. The AAIndex has performed well in protein malonylation site prediction (Chen et al., 2018) and protein ubiquitination site prediction (Tung and Ho, 2008).

This study employed the iLearn platform (Chen et al., 2020) to extract the AAIndex feature. The AAIndex descriptor must be applied to encode peptides of equal length. For the dataset used in this paper, the sequences are unequal. Therefore, to extract an AAIndex feature from the unequal sequence, truncation is required. The new equal sequences, which are used to extract AAIndex features, are obtained by extracting the first ten and last ten letters from the original sequences and then combining them.

### 2.4 Identification model

scikit-learn (Pedregosa et al., 2011) is a mature machine learning algorithm software package based on the Python programming language. It includes a large number of classification, regression, and clustering algorithms and is designed to be used in conjunction with Python’s numerical science libraries NumPy and Scipy. In this paper, we chose a GBDT as our identification algorithm, which is included in the scikit-learn platform. The experimental results are given in Section 3.2.

GBDT is an iterative decision tree algorithm composed of multiple decision trees, with the conclusions of all trees added up to make the final decision. The basic structure of GBDT is a forest of decision trees, and the learning method is gradient upgrading. GBDT has been employed to solve many bioinformatics problems (Gao et al., 2020), such as prognostic biomarker discovery (Li et al., 2022) and Parkinson’s disease prediction (Lee et al., 2022; Yu et al., 2022).

The basic idea behind GBDT is to combine many weak learners to build a strong learner. An individual decision tree acts as one weak learner; all the weak learners are sequentially connected and each tries to minimize the error of the one previous. The sequential connection makes the boosting algorithm learn slowly but more accurately. In general, statistical learning models which learn slowly can perform better.

GBDT generates decision subtrees one by one, thus constructing the whole forest. GBDT generates a new subtree based on the residual of the sample label value and the current forest prediction value. GBDT constructs a strong learner by aggregating the result of each step. The existing trees are not changed when a new tree is added to the forest.

### 2.5 Performance evaluation

The following evaluation indicators are used to demonstrate the performance of our model, including accuracy (ACC), sensitivity (SE), specificity (SP), Mathew’s correlation coefficient (MCC), and the area under the ROC curve (AUC). These evaluation indicators are commonly used in bioinformatics (Zhou et al., 2022; Zhou and Wang, 2022; Chen et al., 2023; Zhang et al., 2023). The calculation formulas of these indicators are as follows:

$$ACC=\frac{TP+TN}{TP+TN+FP+FN},$$ (2)

$$SE=\frac{TP}{TP+FN}$$ (3)

$$SP=\frac{TN}{TN+FP}$$ (4)

$$MCC=\frac{TP\times TN-FP\times FN}{\sqrt{\left(TP+FN\right)\times \left(TP+FP\right)\times \left(TN+FP\right)\times \left(TN+FN\right)}}$$ (5)

where TP (true positive) represents the number of anticancer peptides correctly predicted, FP (false positive) represents the number of non-anticancer peptides predicted as anticancer peptides, TN (true negative) indicates the number of non-anticancer peptides correctly predicted, and FN (false negative) represents the number of anticancer peptides predicted as non-anticancer. SP and SE evaluate the predictive performance of predictors for negative and positive examples, respectively. ACC, MCC, and AUC measure the overall ability of predictors on all examples. The AUC value can be obtained by calculating the area enclosed by the ROC curve. AUC ranges from 0.5 to 1; a larger AUC value indicates that the model is achieving a better and more robust predictive performance.

## 3 Results and discussion

In this section, the performance evaluation of different features is given, under the condition that the classifier is GBDT. Second, the performance evaluation of the classifier is discussed under the condition of using the merged-feature of SVMProt-188D and AAIndex. Finally, the comparison experiment between ACP-GBDT and other methods is given.

### 3.1 Performance of different features

As shown in the framework of our predictor in Figure 1, the merged-features of AAIndex and SVMProt-188D are used to represent the peptide sequences. In this section, we conducted an experiment to prove the validity of our merged-feature using cross-validation on Train-500 and independent testing on Test-164.

GBDT is here employed as the classifier, and the merged-feature is compared with many features based on Train-500, including SVMProt-188D, AAIndex, BLOSUM62 (Lee et al., 2011), CTDD (Dubchak et al., 1995), QSOrder (Chou, 2000), PAAC (Chou and Com, 2001), and some of their combinations. SVMProt-188D encodes peptides as a 188-dimensional vector according to the amino acid composition and the physical and chemical properties of the peptide sequence. AAIndex describes 531 physicochemical properties of amino acids using the AAIndex database. BLOSUM62 uses a matrix (m row and n column) to encode the peptide sequences, where m is set to 20 and n denotes the peptide length. Each row represents the information of one amino acid. CTDD consists of 13 types of physicochemical properties. The 20 amino acids are classified into three categories for each property. CTDD uses five values to represent the peptide sequence of each of the three categories. QSOrder encodes a peptide sequence according to sequence order effect. QSOrder defines a set of sequence-order coupling numbers to represent the sequence-order effect on the condition of the physicochemical distance between amino acids. QSOrder has been successfully applied in protein subcellular location prediction. The full name of PAAC is “pseudo-amino acid composition”; it further extends the amino acid composition with the information of sequence order and successfully predicts the function of rice proteins (Liu et al., 2022).

The experimental results of ten-fold cross-validation on Train-500 are shown in Table 1. Comparing the values of AUC, MCC, and ACC, the merged-feature of AAIndex and SVMProt-188D adopted in this study has a higher value than other feature representation methods. Our merged-feature thus performs better overall. Considering the SE indicator, the value of our merged-feature is still the highest among all methods, demonstrating that our feature representation method can best distinguish anti-cancer peptides from true anti-cancer peptide sequences. Considering the SP indicator, our merged-feature still has a higher value than all the other methods, demonstrating that our method can better identify non-anticancer peptides from negative examples. From all indicators, the merged-featur performance is significantly better than other methods.

**TABLE 1:** Feature comparison on Train-500.

	SE	SP	ACC	MCC	AUC
188D + AAIndex	**90.0**	**90.8**	**90.4**	**81.4**	**94.5**
188D	86.4	86.4	86.4	72.9	92.1
AAIndex	84.8	88.4	86.6	73.7	92.9
BLOSUM62	84.8	90.4	87.6	75.7	92.8
CTDD	83.6	86.4	85.0	70.2	92.2
QSOrder	75.2	79.2	77.2	54.7	85.6
PAAC	76.0	81.6	78.8	58.0	85.3
AAIndex + BLOSUM62	84.8	90.0	87.2	75.0	92.7
CTDD + QSOrder	85.2	87.2	86.2	72.6	92.8
CTDD + PAAC	86.4	85.6	86.0	72.1	92.9
QSOrder + PAAC	76.0	80.4	78.2	56.9	85.9

The maximum values in the table are bold.

In order to verify the generalization performance of using the merged-feature, our merged-feature is compared with other features on the independent test dataset Test-164. As shown in Table 2, according to SE, SP, AUC, MCC, and ACC, the merged-feature achieves the highest value of all feature representation methods, including single feature and some combinational features. The merged-feature SVMProt-188D/ AAIndex performs better than single SVMProt-188D or single AAIndex. Therefore, it verified that performance can be improved by combining the different features. Comparing the identification performance of six single-features in Table 2, AAIndex achieves the highest value of all evaluation indicators. The physicochemical properties of amino acids can thus better identify ACPs.

**TABLE 2:** Feature comparison on Test-164.

	SE	SP	ACC	MCC	AUC
188D + AAIndex	**90.2**	**92.7**	**91.5**	**83.0**	**95.6**
188D	84.1	84.1	84.1	68.3	92.8
AAIndex	85.4	**92.7**	89.0	78.3	92.9
BLOSUM62	85.4	89.0	87.2	74.4	92.8
CTDD	84.1	81.7	82.9	65.9	91.7
QSOrder	80.5	76.8	78.7	57.4	88.3
PAAC	78.0	79.3	78.7	57.3	86.3
AAIndex + BLOSUM62	85.4	**92.7**	89.0	78.3	93.0
CTDD + QSOrder	85.4	80.5	82.9	65.9	92.3
CTDD + PAAC	85.4	86.6	86.0	72.0	91.7
QSOrder + PAAC	78.0	79.3	78.7	57.3	88.1

The maximum values in the table are bold.

By considering the ten-fold cross-validation on Train-500 and independent test verification on Test-164, we find that the merged-feature best recognizes ACPs.

### 3.2 Performance of different classifiers

We chose GBDT as the classifier to identify anticancer from peptide sequences. To verify the validity of the classifier GBDT, the performance of GBDT is compared with other classifiers on Train-500 and Test-164. The classifiers used for comparison include XGBoost, random forest, AdaBoost, decision tree, k-nearest neighbor, logistic regression, and GaussianNB. eXtreme Gradient Boosting (XGBoost) is an implementation of a boosting algorithm. It is a meta-algorithm that can integrate weak classifiers to build strong classifiers (Schaduangrat et al., 2019; Prabha et al., 2021). XGBoost is essentially a GBDT. Compared with the GBDT algorithm, XGBoost maximizes speed and efficiency. Random forest (RF) is an effective machine learning algorithm (Ao et al., 2022b; Tran and Nguyen, 2022; Naik et al., 2023) which is a random composition of many unrelated decision trees. When judging the category of a new sample, each RF decision tree makes an independent judgment and finally selects the category with the highest probability value. Adaptive Boosting (AdaBoost) (Rojas, 2009) is an adaptable boosting algorithm that changes the weights of samples classified by a prior basic classifier. Samples with new weights are then input into a new weak classifier to produce enhanced training results. Finally, a strong classifier will be obtained when the minimum error rate or the maximum number of iterations is reached. The decision tree classification algorithm constructs a tree-type classification model from the training samples (Shabbir et al., 2021). The decision nodes (non-leaf nodes) in the tree are used to judge the category, and each leaf node represents the classification of the sample. For a new sample, the decision tree can give a path from the root node to some leaf nodes, with the latter representing the prediction category of the sample. The K-Nearest Neighbors (KNN) (Kramer, 2013) classification algorithm is very simple: it determines the category of the target point by “voting” through the category of n points closest to the target point. The algorithm stores the learned data and classifies unseen data by computing its distance with training data. Logistic regression is actually a classification method that assumes that the data are subject to Bernoulli distribution and solves the parameters by the maximum likelihood function and gradient descent methods to achieve data classification (Ksiazek et al., 2021). Gaussian Naive Bayes (GaussianNB) (Bong and Kim, 2022) is a naive Bayes with *a priori* Gaussian distribution; the conditional probability of each characteristic dimension of the sample is assumed to obey Gaussian distribution, the posterior probability of each category of the new sample under a certain characteristic distribution is then calculated according to the Bayes formula, and finally, the category of the sample is determined by maximizing the posterior probability.

Using the merged-feature of SVMProt-188D and AAIndex as the feature representation of the anticancer peptide sequences, the ten-fold cross-validation results of the GBDT and the other classifiers on Train-500 are shown in Table 3. GBDT’s value is higher than the other classifiers in ACC, MCC, and AUC indicators. GBDT thus outperforms the other classifiers on the anticancer peptide dataset. Considering the indicator SE, the value of GBDT is still higher than all the other classifiers, meaning that it performs best in identifying anti-cancer peptides in peptide sequences. For the SP indicator, the value of GBDT is slightly lower in RF but higher in the SE indicator, meaning that RF is more biased than GBDT in classifying an unseen peptide as negative sample. GBDT especially achieves higher values than RF for the ACC, MCC, and AUC indicators; GBDT thus has better overall performance than RF. Therefore, in general, the GBDT classifier has better recognition capability.

**TABLE 3:** Classifier comparison on Train-500.

	SE	SP	ACC	MCC	AUC
GBDT	**90.0**	90.8	**90.4**	**81.4**	**94.5**
XGBoost	87.2	88.8	88.0	76.4	94.3
RandomForest	87.2	**92.0**	89.6	79.8	94.4
AdaBoost	82.4	81.6	82.0	64.4	89.6
DecisionTree	87.6	81.6	84.6	70.0	84.6
Kneighbors	82.4	74.8	78.6	57.7	87.1
LogisticRegression	75.6	72.4	74	48.1	79.4
GaussianNB	74.8	80.8	77.8	56.1	80.6

The maximum values in the table are bold.

To verify the generalization performance, the GBDT adopted in this paper is compared with the other seven classifiers on the independent test dataset Test-164; the result is shown in Table 4. We found that GBDT achieves the best value among all classifiers on all indicators. The accuracy of using GBDT reaches a maximum value of 91.5%. Therefore, the GBDT classifier better generalizes anticancer datasets.

**TABLE 4:** Classifier comparison on Test-164.

	SE	SP	ACC	MCC	AUC
GBDT	**90.2**	**92.7**	**91.5**	**83.0**	**95.6**
XGBoost	89.0	**92.7**	90.9	81.8	94.9
RandomForest	84.1	91.5	87.8	75.8	94.2
AdaBoost	85.4	**92.7**	89	78.3	93.6
DecisionTree	80.5	86.6	83.5	67.2	83.5
Kneighbors	81.7	64.6	73.2	47.0	81.3
LogisticRegression	85.4	83.0	84.1	68.3	88.6
GaussianNB	73.2	65.9	70.0	39.1	72.8

The maximum values in the table are bold.

### 3.3 Comparison with other methods

To measure the effectiveness of ACP-GBDT, we compared it with the other eight existing methods (Wu et al., 2022)—ACP-MCAM, ACP-Fuse, AntiCP_ACC, AntiCP_DC, PEPred-Suite, ACPred-FL, iACP, and Hajisharifi’s method—on Train-500 and Test-164. The ten-fold cross-validation results on Train-500 are given in Table 5. The independent test results on Test-164 are given in Table 6.

**TABLE 5:** Comparison with state-of-the-art methods on Train-500.

	SE	SP	ACC	MCC	AUC
ACP-GBDT	**90.0**	90.8	**90.4**	**81.4**	**94.5**
iACP	57.2	84.0	70.6	42.8	80.9
ACPred-FL	71.6	84.4	78.0	56.5	84.6
PEPred-Suite	72.8	88.0	80.4	61.5	86.0
ACPred-Fuse	77.2	87.6	82.4	65.2	88.2
AntiCP_ACC	66.8	78.4	72.6	45.5	82.4
AntiCP_DC	71.6	77.6	74.6	49.3	82.5
Hajisharifi’s	67.2	83.6	75.4	51.5	83.1
ACP-MCAM	85.6	**95.2**	90.4	81.3	91.9

The maximum values in the table are bold.

**TABLE 6:** Comparison with state-of-the-art methods on Test-164.

	SE	SP	ACC	MCC	AUC
ACP-GBDT	**90.2**	92.7	**91.5**	**83.0**	**95.6**
iACP	54.9	88.8	87.7	22.6	76.1
ACPred-FL	69.5	85.8	85.3	25.9	85.1
PEPred-Suite	68.3	90.6	89.9	32.0	86.1
ACPred-Fuse	72.0	89.5	89.0	32.0	86.8
AntiCP_ACC	68.3	88.5	87.9	28.8	85.3
AntiCP_DC	68.3	82.6	82.2	22.3	83.0
Hajisharifi’s	69.5	88.4	87.9	29.2	85.5
ACP-MCAM	85.4	**96.3**	90.9	82.2	94.8

The maximum values in the table are bold.

In Table 5, the ACP-GBDT predictor has a larger value than other methods in the AUC, ACC, and MCC indicators, reaching 90.4%, 81.4%, and 94.5%, respectively. The ACP-GBDT predictor thus performs better overall than other state-of-art methods. For SP, the value of our method is higher than the other methods except ACP-MCAM. For SE, our method is higher than the other methods, meaning that it best identifies anti-cancer peptides from positive samples. Comprehensively considering SP and SE, ACP-MCAM is biased to classifying an example as negative compared with ACP-GBDT. Therefore, ACP-GBDT performs better than the other predictors.

In Table 6, the identification ability of the ACP-GBDT predictor is superior to the other methods based on the AUC, MCC, and ACC indicators, reaching 91.5%, 83.0%, and 95.6%, respectively.

For SE and SP, the ACP-GBDT predictor has a higher value than the other methods except ACP-MCAM, which means that our method better classifies an unseen sample than other methods, except ACP-MCAM.

Although ACP-MCAM has a higher value than ACP-GBDT on SP, its SE value is lower than our method. ACP-MCAM is thus biased toward classifying an example as a non-anticancer peptide. In general, experimental results on an independent test dataset demonstrated that our predictor is superior to the others for anticancer identification.

## 4 Conclusion

In this study, we developed a simple and effective anticancer peptide predictor and named it “ACP-GBDT.” Considering the physicochemical properties and composition of amino acids, a feature merge of AAIndex and SVMProt-188D was built. GBDT was employed to classify anticancer and non-anticancer. The experimental test on features and classifiers has been given. The feature experiments show that the performance of merged-feature is better than that of single-feature and their combinations. The classifier experiments on the independent test set and ten-fold cross-validation set show that the GBDT classifier performs better than other classifiers. In the future, we plan to add a feature selection strategy.

## Data Availability

Publicly available datasets were analyzed in this study. These data can be found at https://github.com/jingry/autoBioSeqpy/tree/master/examples/anticancer_peptide_prediction/data.
